# Upper respiratory tract microbiota is associated with small airway function and asthma severity

**DOI:** 10.1186/s12866-023-02757-5

**Published:** 2023-01-13

**Authors:** Yi Li, Congying Zou, Jieying Li, Wen Wang, Yue Guo, Lifang Zhao, Chunguo Jiang, Peng Zhao, Xingqin An

**Affiliations:** 1 State Key Laboratory of Severe Weather of CMA, Chinese Academy of Meteorological Sciences, Beijing, 100081 China; 2 Department of Surgery, Beijing ChaoYang Hospital, Capital Medical University, Chaoyang District, Beijing, China; 3 Department of Respiratory and Critical Care Medicine, Beijing Institute of Respiratory Medicine and Beijing Chao-Yang Hospital, Capital Medical University, No.8, Gongtinan Road, Chaoyang District, Beijing, 100020 China; 4 Department of Health and Environmental Sciences, Xi’an Jiaotong-Liverpool University, Suzhou, China

**Keywords:** Ashtma, Mirobiome, Phenotype, Small airway function, Maximal expiratory flow

## Abstract

**Background:**

Characteristics of airway microbiota might influence asthma status or asthma phenotype. Identifying the airway microbiome can help to investigate its role in the development of asthma phenotypes or small airway function.

**Methods:**

Bacterial microbiota profiles were analyzed in induced sputum from 31 asthma patients and 12 healthy individuals from Beijing, China. Associations between small airway function and airway microbiomes were examined.

**Results:**

Composition of sputum microbiota significantly changed with small airway function in asthma patients. Two microbiome-driven clusters were identified and characterized by small airway function and taxa that had linear relationship with small airway functions were identified.

**Conclusions:**

Our findings confirm that airway microbiota was associated with small airway function in asthma patients.

**Supplementary Information:**

The online version contains supplementary material available at 10.1186/s12866-023-02757-5.

## Background

Asthma is a heterogeneous disease characterized by inflammation and hyperresponsive in airways, which has several phenotypes and endotypes that may response differently to therapies. Despite important advances in asthma, including greater awareness, timely diagnosis, and pharmacological interventions targeted at airway inflammation, control of asthma in patients remains unsatisfactory.

A possible reason for poor asthma control might be that other than “Eosinophils asthma phenotype” or “Neutrophil asthma phenotype”, some patients express a “small airways phenotype”, which has small airways inflammation and dysfunction that is not being targeted or controlled by current therapies. The small airways are defined by an internal airway diameter of < 2 mm. They have a generation number that is generally higher than 8, and they account for 98.8% (approximately 4500 ml) of the total lung volume, compared to that the large airways account for only 1.2% (approximately 50 ml). Though inflammation and remodeling in asthma involve the large airways, the small airways are the major site of airflow limitation, and where the intensity of the inflammation may be even higher than that in large airways. Transbronchial biopsy findings show that small airways are the major site of inflammation and contain immunocytes that putatively account for the tissue remodeling noted [1, 2]. Thus, small airways might affect the pathobiology of asthma and small airway dysfunction may contribute to poor asthma control [1–4], and the small airways of individuals with asthma are increasingly recognized as a potential therapeutic target [2, 4, 5].

The microbiota in human airways changes with disease. With the bacterial 16S ribosomal RNA gene sequencing technique, different microbiota were identified between asthma phenotypes, suggesting that microbial patterns in the airways may influence distinct phenotypes of asthma [6–8] and allergic inflammation [9]. Airway microbiota composition is also associated with the degree of airway hyperresponsiveness among patients with less controlled asthma. Indeed, several bacterial taxa, including *Streptococcus pneumonia*, *Staphylococcus aureus*, *Moraxella catarrhalis*, *Pseudomonas aeruginosa*, and *Haemophilus influenza*, were reported to be associated with asthma exacerbation or development [10, 11]. Moreover, studies suggest that airway microbiome in asthma patients is probably a result of complex interactions between the inflammatory milieu and the drug effects, and microbial-derived mechanisms might be the reason of poor response to the treatment. For example, treatment with a combination of inhaled corticosteroids (ICSs) and oral glucocorticoids correlates positively with an increased abundance of *Proteobacteria* and *Pseudomonas*, and with a decreased abundance of *Bacteroidetes*, *Fusobacteria*, and *Prevotella* [12]. Meanwhile, a unique enrichment of *Haemophilus*, *Neisseria*, *Fusobacterium*, *Porphyromonas* species and the *Sphingomonodaceae* family along with depletion in *Mogibacteriaceae* and *Lactobacillales* was observed in mild asthma patients without being treated with ICSs [13].

In this study, the association between airway microbiota pattern and small airway function was explored. Results from lung function tests were related to the bacterial flora in study subject sputum.

## Methods

### Pulmonary function measurements

The measurements of spirometry function were conducted by Jaeger Masterscreen PFT (Viasys Healthcare, Höchberg, Germany) according to the recommendations of the *Chinese National Guidelines of Pulmonary Function Test* [14]. Following indices were used to characterize small airway function: forced expiratory volume in first second (FEV1), forced expiratory vital capacity (FVC), peak expiratory flow (PEF), maximal expiratory flow at 25% vital capacity (MEF25), maximal expiratory flow at 50% vital capacity (MEF50), percentage of tested MEF25 to predicted MEF25 (MEF25_pred%_), percentage of tested MEF50 to predicted MEF50 (MEF50 _pred%_) and forced expiratory flow between 25 and 75% (MEF (75/25)).

### Study population

All individuals with asthma were patients from the Respiratory Department in Chaoyang Hospital, Beijing, while 12 healthy individuals were recruited from routine physical examination department in the same institution. The age distribution of these healthy people were from 28 to 58 and they were ruled out of asthma and other respiratory diseases by scan examination and pulmonary function tests according to the *Global Strategy for Asthma Management and Prevention* [15, 16].

Among the 31 individuals with asthma, we took a cut-off value of 65% for MEF25_pred%_ and MEF50 _pred%_ to define study groups according to the Chinese Thoracic Society [17–19]. We defined patients who had a MEF25_pred%_ lower than 65% as the MEF_25pred%_-low group (26 people), and others with a MEF_25pred%_ value higher than 65% as the MEF_25pred%_-high group (5 people). The MEF50_pred%_-low group and MEF50_pred%_-high group were similarly defined, and 14 patients were grouped in the MEF50_pred%_-high group versus 17 in the MEF50_pred%_-low group.

As MEF50 and MEF25 are similar indices of small airway function, and because the sample size of the MEF50pred%-high group and MEF50_pred%_-low group is closer than those of MEF25_pred%_ groups, we compared the sputum microbiome only between the MEF50 groups and the healthy individuals.

Subject characteristics are presented in Table 1.

## Sampling of induced sputum

Induced sputum from asthma patients and health individuals was collected according to standardized protocols [20, 21]. Study subjects were pre-treated with inhaled salbutamol to relax airway smooth muscle and to prevent acute asthma attack. Then they inhaled a nebulized solution of 3% saline over a 2-minute period, spat out the saliva, took 2 deep inspirations of saline, and coughed sputum into a separate cup. This procedure was repeated for six times. Subjects were instructed to rinse orally with water and to blow their nose after each inhalation to avoid contamination with saliva and post-nasal drip. Sputum samples were collected into sterilized pots and stored at − 80 °C for bacterial DNA extraction. Peak flow is monitored throughout the procedure, if patients feel uncomfortable or symptoms occurred, the induction was stopped.

## DNA extraction, PCR amplification and Illumina sequencing

Microbial DNA was extracted from induced sputum. The final DNA concentration and purification were determined by NanoDrop 2000 UV-vis spectrophotometer (Thermo Scientific, Wilmington, USA), and DNA quality was checked by 1% agarose gel electrophoresis. The V3-V4 hypervariable regions of the bacteria 16S rRNA gene were amplified with primers 338F (5′- ACTCCTACGGGAGGCAGCAG-3′) and 806R (5′-GGACTACHVGGGTWTCTAAT-3′) by thermocycler PCR system (GeneAmp 9700, ABI, USA). The PCR reactions were conducted using the following program: 3 min of denaturation at 95 °C, 27 cycles of 30 s at 95 °C, 30s for annealing at 55 °C, and 45 s for elongation at 72 °C, and a final extension at 72 °C for 10 min. PCR reactions were performed in triplicate 20 μL mixture containing 4 μL of 5 × FastPfu Buffer, 2 μL of 2.5 mM dNTPs, 0.8 μL of each primer (5 μM), 0.4 μL of FastPfu Polymerase and 10 ng of template DNA. The resulted PCR products were extracted from a 2% agarose gel and further purified using the AxyPrep DNA Gel Extraction Kit (Axygen Biosciences, Union City, CA, USA) and quantified using QuantiFluor™-ST (Promega, USA) according to the manufacturer’s protocol.

Purified amplicons were pooled in equimolar and paired-end sequenced (2 × 300) on an Illumina MiSeq platform (Illumina, San Diego, USA) according to the standard protocols [22, 23].

## Bioinformatics analysis

The analysis was conducted by following the “Atacama soil microbiome tutorial” of Qiime2docs along with customized program scripts (https://docs.qiime2.org/2019.1/). Briefly, raw data FASTQ files were imported into the format which could be operated by QIIME2 system using qiime tools import program. Demultiplexed sequences from each sample were quality filtered and trimmed, de-noised, merged, and then the chimeric sequences were identified and removed using the QIIME2 dada2 plugin to obtain the feature table of amplicon sequence variant (ASV). The QIIME2 feature-classifier plugin was then used to align ASV sequences to a pre-trained GREENGENES 13_8 99% database (trimmed to the V3V4 region bound by the 338F/806R primer pair) to generate the taxonomy table. Any contaminating mitochondrial and chloroplast sequences were filtered using the QIIME2 feature-table plugin.

Experimental materials and reagents are included in supplementary material (suppl. Table 1).

## Statistics and identification of bacterial communities

We used a rank test method, the Kruskal–Wallis test to examine the differences between groups. The linear Discriminant Analysis Effect Size (LEfSe) method [24] was employed to compare the bacterial composition between groups, with the cutoff *p*-value set as 0.05 (after Benjamini-Hochberg false discovery rate correction). Additionally, Kyoto Encyclopedia of Genes and Genomes (KEGG) functional profiles of microbial communities were predicted with Phylogenetic Reconstruction of Unobserved States (PICRUSt) [25].

Microbiome Multivariable Associations with Linear Models (MaAsLins) were run to test for associations between microbiomes and clinical variables using the MaAsLin 2 R/Bioconductor software package [26, 27]. The linear mixed-effect model could be expressed as follows:

$$Bacterial\;taxon\:\sim\:(intercept)\:+\:small\;airway\;index\:+\:(\mathit1\;/subject).$$

All analyses were performed using R studio (version 1.1.453) [28] with R software (version 3.5.1) [29] supported with the following software packages: vegan, metacoder [30], MaAsLin2 [27], ggplot2, Tax4Fun2 [31], and mixOmics [32].

## Results

### Clinical characteristics of the study subjects

Clinical features of the subjects are shown in Table 1, all significantly (*p* < 0.05) different indices between MEF25_pred%_-high group and MEF25 _pred%_-low group or between MEF50_pred%_-high group and MEF50 _pred%_-low group were marked with a “*”. As expected, FEV_1_, FEV_1_/FEC, MEF25, MEF50, MEF75 and MEF (75/25) values were significantly (*p* < 0.05) lower in MEF25_pred%_-low group than those in MEF25 _pred%_-high group, meanwhile neutrophil and Fractional exhaled nitric oxide (FeNO) were significantly (*p* < 0.05) higher in in MEF25_pred%_-low group than those in MEF25 _pred%_- high group. VC, PEF, FEV1, FEV1/FEC, MEF25, MEF50, MEF75 and MEF (75/25) values were significantly (*p* < 0.05) lower in MEF50_pred%_-low group than those in MEF50 _pred%_-high group. Associations between these significantly different indices and microbiome were investigated by MaSlin2 (in later sections).

**Table 1:** Clinical characteristics of study subjects^a^

	MEF_25pred%_		MEF_50pred%_	
	high	low	high	low
Subject(n)	5	26	14	17
Age	34.40 ± 16.20	46.34 ± 12.83	41.35 ± 14.72	46.94 ± 13.00
Male (%)	60	54	57	47
Atopy (%)	100	85	90	84
BMI^#^	24.61 ± 5.66	26.74 ± 4.54	25.43 ± 4.12	27.19 ± 5.11
MEF25	(1.9 ± 0.81)*	(0.69 ± 0.36)*	(1.34 ± 0.65)**	(0.51 ± 0.29)**
MEF50	(4.33 ± 1.32)*	(2.19 ± 1.16)*	(3.73 ± 1.05)**	(1.55 ± 0.74)**
MEF75	(8.02 ± 2.45)*	(4.49 ± 2.25)*	(7.22 ± 1.86)**	(3.28 ± 1.54)**
MEF(75/25)	(3.85 ± 1.28)*	(1.7 ± 0.89)*	(3.06 ± 1.02)**	(1.22 ± 0.62)**
FeNO (ppb)	(24.8 ± 12.38)*	(41.32 ± 24.66)*	30.86 ± 14.44	45.31 ± 28.38
IgE/(ng·mL^− 1^)^b^	148.9 ± 71.55	326.48 ± 393.57	277.93 ± 430.54	319.11 ± 340.12
Neutrophil^b^ (× 10^9^/L)	(3.27 ± 0.32)**	(5.06 ± 1.40)**	4.29 ± 1.71	5.16 ± 1.21
Eosinophils^b^ (× 10^9^/L)	0.34 ± 0.33	0.51 ± 0.65	0.31 ± 0.28	0.59 ± 0.74
ACT score	23 ± 2.65	21.43 ± 3.81	23 ± 1.89	20.75 ± 4.3
AQLQ score	82.67 ± 17.5	88.27 ± 15.1	90.2 ± 12.4	85.87 ± 16.88
VC	4.18 ± 0.75	3.66 ± 0.97	(4.13 ± 0.75)*	(3.43 ± 0.99)*
FVC	4.15 ± 0.75	3.64 ± 0.98	(4.11 ± 0.76)*	(3.42 ± 1.01)*
FEV1	(3.58 ± 0.75)*	(2.51 ± 0.85)*	(3.32 ± 0.66)**	(2.15 ± 0.75)**
FEV1/FVC	(102.88 ± 7.78)**	(81.12 ± 13.07)**	(97.01 ± 6.52)**	(74.45 ± 11.3)**
PEF	8.98 ± 2.07	7.01 ± 2.21	(8.71 ± 1.96)**	(6.17 ± 1.87)**

^a^Data are expressed as mean values and standard errors

^b^counts in blood

*: indicating a statistical significant difference between MEF25_pred%_-high and MEF25_pred%_-low groups, or between MEF50_pred%_-high and MEF50_pred%_-low groups. Significant level: *, *p* < 0.05; **: *p* < 0.01

No significant difference of blood eosinophils or serum IgE was observed between these two pairs of groups.

### Sputum microbiome compositions

A total of 2,305,983 valid reads were generated for the 43 samples. After filtering for low-quality reads, 51,245 sequence reads were used for subsequent analyses and resulted in 12,265 OTUs. The average percentage of input passed filter was approximately 85%, and average percentage of input non-chimeric was approximately 77%.

**Fig. 1:**
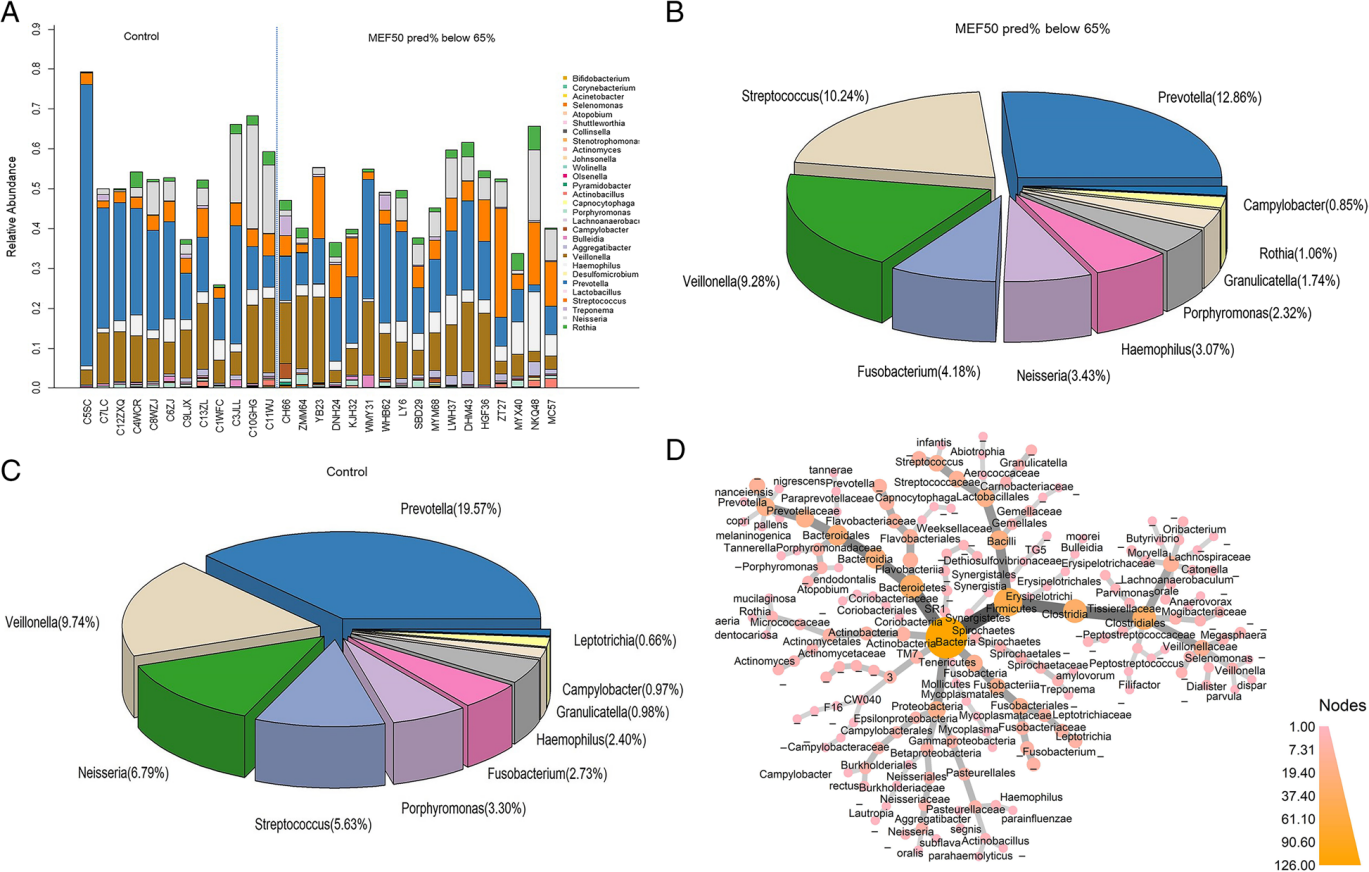
The sputum microbiome at the genus level. **A** Bar plot of all the samples, each bar shows the relative abundance of one individual **B**) Pie chart of the microbiome composition at genus level in MEF50_pred%_-low group. **C** Pie chart of the microbiome composition at genus level in healthy individuals. **D** Phylogenetic map of the median relative abundance differences in bacterial taxa between the healthy control group and the MEF50_pred%_-low group, the ending circle of each branch represented for species (*n* = 29). The depth of color of the nodes corresponds to the degree of difference in median relative abundance of the bacterial taxa. The darker the color of the phylogenetic branches, the higher median differences, whereas gray nodes and branches indicate no significant differences

We first examined the sputum microbiome composition. Taxa barplots and pie chart of bacterial genera in healthy control subjects and MEF50_pred%_-low group are presented in Fig. 1A, B and C . At the genus level, the top five genera of the healthy control sputum microbiome were *Prevotella* (19.57%), *Veillonella* (9.74%), *Neisseria* (6.80%), *Streptococcus* (5.63%), *Porphyromonas* (3.30%). The top five genera of MEF50_pred%_-low group was *Prevotella* (12.86%), *Streptococcus* (10.24%), *Veillonella* (9.27%), *Fusobacterium* (4.18%) and *Neisseria* (3.43%) .

We then and compared the difference in the median relative abundance of taxa between the healthy individuals and MEF50_pred%_-low group, as the metagenomics phylogenetic map shows in Fig. 1D.

It could be seen from Fig. 1D that the largest significant (*p* < 0.05) difference in the median relative abundance of taxa was observed in the genus *Prevotella*, which was in accordance with the difference in microbiome composition. At the species level in this genus, significant (*p* < 0.05) difference was observed in species *Prevotella nanceiensis* (*P. nanceiensis*), *Prevotella nigrescens (P. nigrescens)*, *Prevotella copri (P. copri)* and *Prevotella pallens (P. pallens),* and all these species had a relative abundance higher than 0.01% (Supplementary Fig. 1).

The second largest significant (*p* < 0.05) difference in the median relative abundance of taxa was observed in genus *Streptococcus*. At the species level in this genus, the relative abundance of *Streptococcus infantis (S. infantis)* was significantly different between MEF50_pred%_-low group and healthy control group (Supplementary Fig. 1).

Other species that had significant (*p* < 0.05) difference in relative abundance between MEF50_pred%_-low group and the healthy controls include *Campylobacter rectus* (*C. rectus*) and *Collinsella aerofaciens (C. aerofaciens)* (Supplementary Fig. 1).

### Partial least squares discriminant analysis (PLS-DA) of microbial difference

**Fig. 2:**
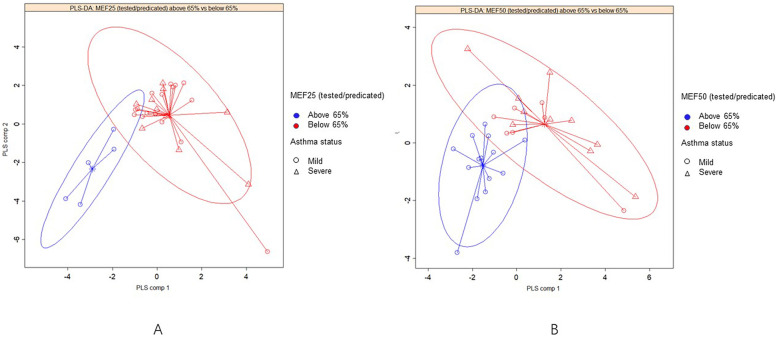
Supervised PLS-DA plots with confidence ellipse, arrows point to the outcome category of each subject, including mild asthma and severe asthma as a subgroup. **A** MEF25 _pred%_ group with a subgroup of asthma status. **B** MEF50 _pred%_ group with a subgroup of asthma status

The PLS-DA model was established to identify the contribution of taxa to the difference in the community structure between the groups. Figure 2 shows the results of supervised PLS-DA plots concerning the microbial difference between MEF25 _pred%_ and MEF50 _pred%_ functional groups. It could be seen that the two clusters were characterized by composition difference according to MEF25 _pred%_ (Fig. 2A), MEF50 _pred%_ (Fig. 2B) function and asthma severity.

**Fig. 3:**
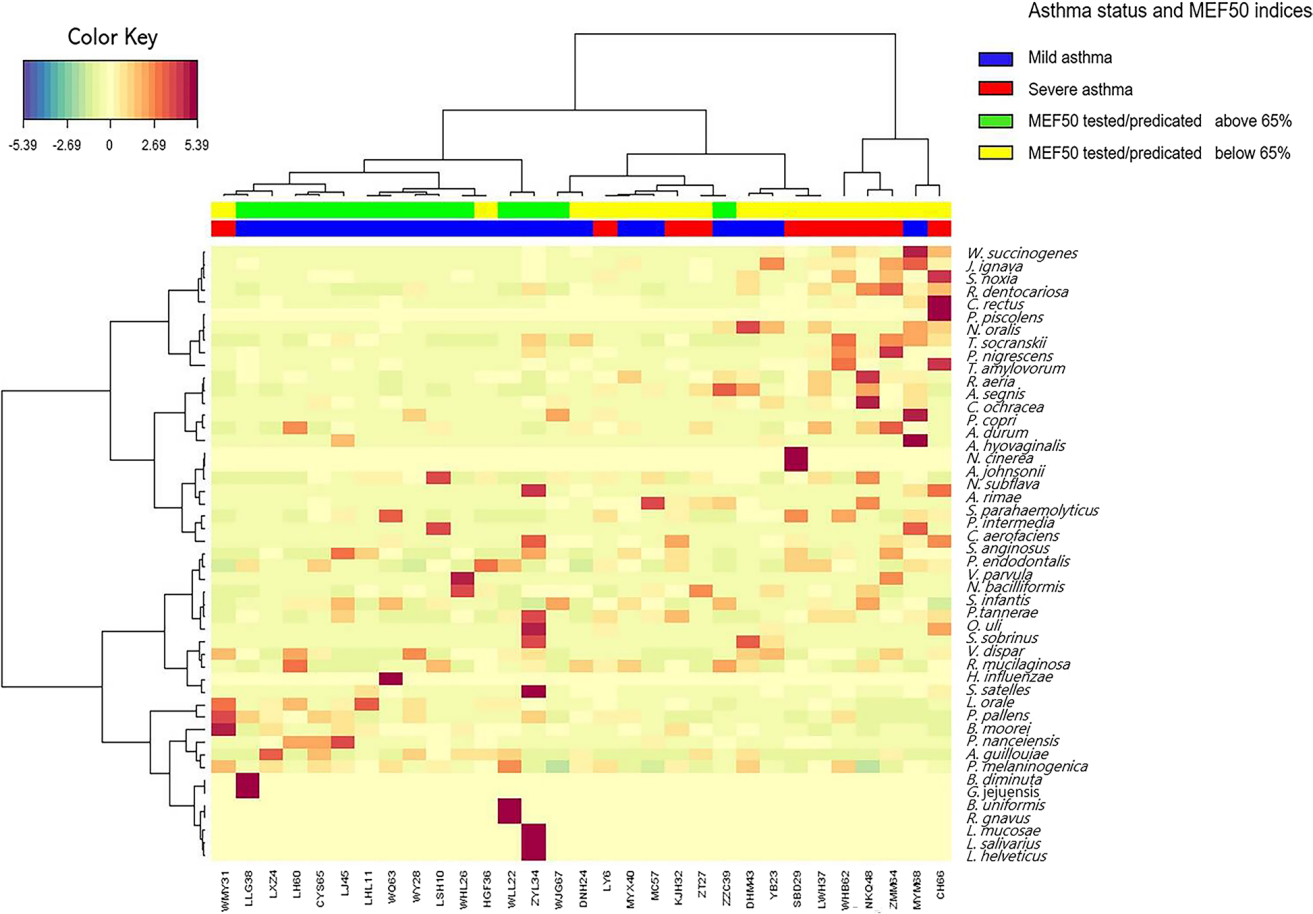
Clustered image maps by different MEF50 _pred%_ groups, including asthma status as a subgroup. Samples are represented in columns and taxa in rows. The colored side at the top of the heatmap indicates different groups. (Note: this plot was created with package mixOmics [32] of R software (version 3.5.1) [29])

Moreover, a heat map of the Euclidian distance of taxa between clusters characterized by MEF50 _pred%_ function groups was shown in Fig. 3, indicating the distribution of taxa to component 1 in each sample.

It could be seen that in the MEF50_pred%_-low group, at the species level, *Johnsonella ignava*, *Rothia dentocariosa* (*R. dentocariosa*), *C. rectus*, *Treponema socranskii* (*T. socranskii*), *P. nigrescens*, *Treponema amylovorum*, *Aggregatibacter segnis* (*A. segnis*), and *Corynebacterium durum* had the largest Euclidian distance between the two clusters. Meanwhile, in the MEF50_pred%_-high group, *Veillonella dispar*, *P. pallens*, *P. nanceiensis*, and *P. melaninogenica* had the largest Euclidian distance between the two clusters.

### Linear associations between sputum microbiome and small airway indices

**Fig. 4:**
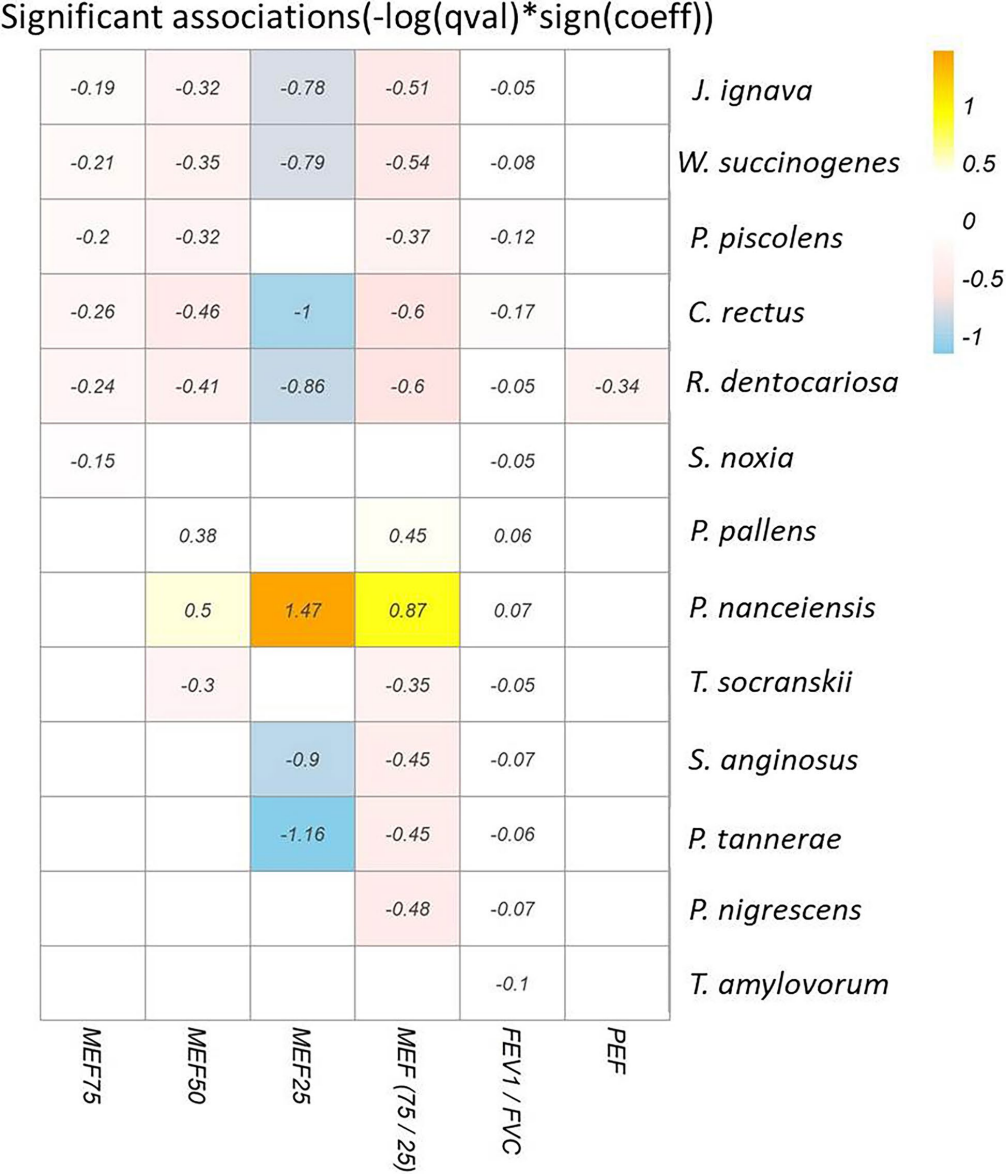
MaAslin analysis of the heat map between small airway indices and microbiome. Only significant (*p* < 0.05) associations are shown. The numbers in the figure indicate the magnitude of coefficients in the linear associations

Mixed multiple linear regression analysis (MaAslin) was performed to explore whether there was a linear relationship between sputum microbiomes with MEF25, MEF50, MEF75, PEF, MEF (75/25), and FEV_1_/FVC. Figure 4 shows the heat map of these significant (*p* < 0.05) estimates, indicating the magnitude of coefficients in the linear associations.

It could be seen that MEF (75/25) and FEV_1_/FVC had most associations with the microbiome. Only two species, *P. nanceiensis* and *P. pallens*, had positive associations with MEF25, MEF50, MEF (75/25), and FEV_1_/FVC levels, whereas the species *J. ignava*, *R. dentocariosa*, *W. succinogenes*, and *C. rectus* had negative associations with the three MEF indices. The species *P. piscolens* had a negative association with MEF25, MEF50, MEF (75/25), and FEV_1_/FVC levels. The species *Selenomonas noxia* had a negative association with MEF75 and FEV1/FVC*,* while the species *T. socranskii* had a negative association with MEF50, MEF75/25, and FEV_1_/FVC. Lastly, the species *Streptococcus. anginosus* and *Prevotella tannerae* had negative associations with MEF25, MEF75/25, and FEV_1_/FVC.

### KEGG pathway analysis

**Fig. 5:**
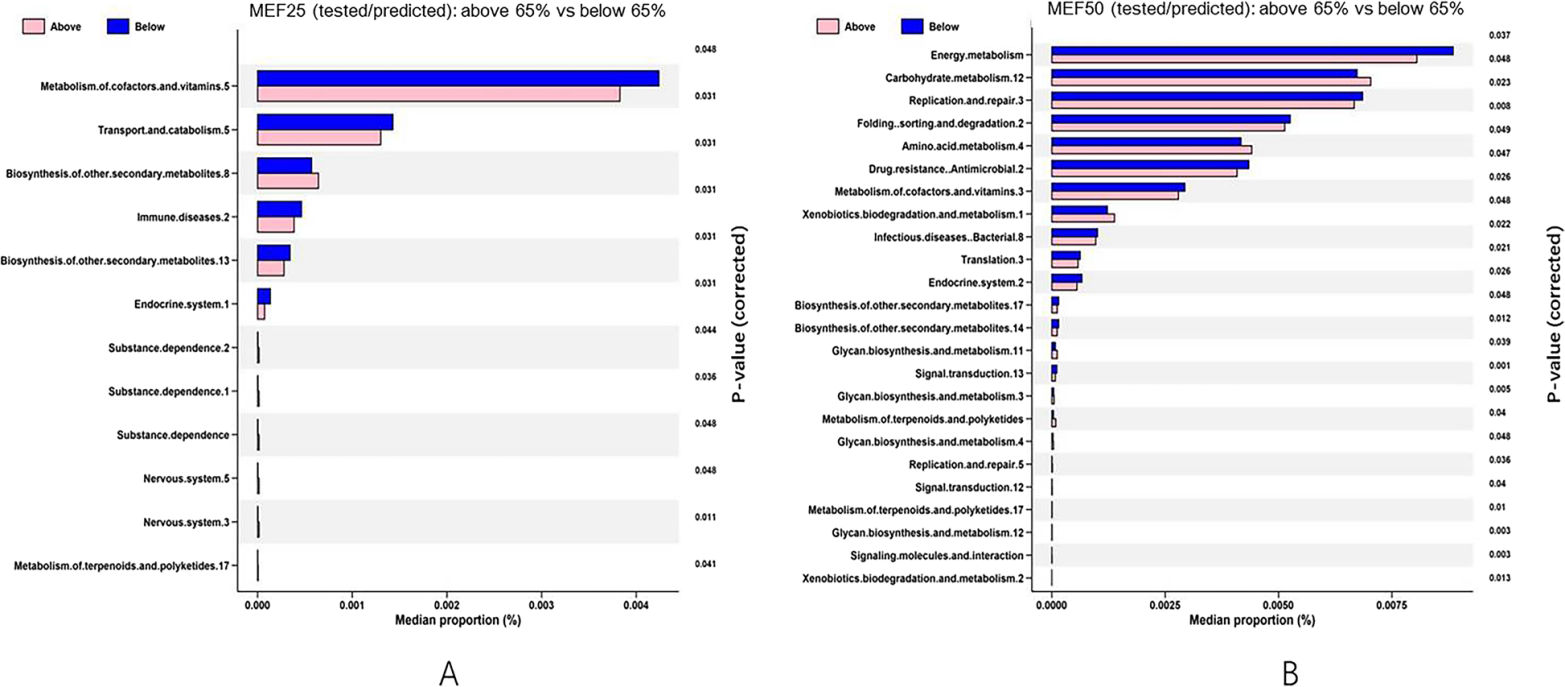
KEGG pathway analysis between the study groups. **A** MEF25_pred%_-low and MEF25 _pred%_-high groups. **B** MEF50_pred%_-low and MEF50 _pred%_-high groups. (Note: all the KEGG identifiers were from https://www.kegg.jp/kegg/)

16S rDNA amplicon data were supplemented with genomicdata using PICRUSt. Genes from different bacteria likely to perform the same function have been grouped into KEGG orthologues (KO) by the Kyoto Encyclopedia of Genes and Genomes (KEGG). Differentially abundant KOs were screened by using the Bonferroni-corrected Wilcoxon rank sum test for differences between healthy individuals and MEF50_pred%_-low group. Significant (*p* < 0.05) differences are shown in Fig. 5. In the MEF25_pred%_-low and MEF25 _pred%_-high groups, changes in the microbial flora of function genes in six categories were related to pathways associated with metabolism of cofactors and vitamins, transport and catabolism, biosynthesis and secondary metabolism, immune disease, and the endocrine system (Fig. 5A). For the MEF50_pred%_-low and MEF50-high groups, changes in the function genes were in genes associated with energy and carbohydrate metabolism, replication and repair, protein folding, sorting and degradation, amino acid metabolism, drug resistance, xenobiotics, and infectious disease (Fig. 5B).

## Discussion

In this study, we found significant differences in the composition, relative abundance, biomarkers and signaling pathways of airway microbiome between small airway functional groups and healthy controls. Two microbiome-driven clusters were identified and characterized by small airway function, and change in the microbiome composition between small airway functional group was observed. Our study gave evidence to the connection between respiratory tract microbiota and small airway function in asthma patients.

Although the precise role of bacterium in airway inflammation remains to be established, some genera or bacteria were reported to be associated with asthma severity and phenotype. Specifically, genera *Haemophilus*, *Moraxella*, and *Neisseria* of the phylum *Proteobacteria*, or species *Haemophilus influenzae* and *Moraxella catarrha*, were associated with worse asthma control [6, 8, 33, 34]. In this study, we also found some associations between specific bacteria and small airway functions. First, we observed two species, *P. pallens* and *P. nanceiensis*, were correlated with better small airway function and better asthma status.

These two species had positive linear estimates with MEF50, MEF25, MEF (75/25) and FEV_1_/FVC. More than that, *P. nanceiensis* was a biomarker in the healthy control group (Supplementary Fig. 2), and its relative abundance significantly (*p* < 0.05) decreased in small airway dysfunction groups (MEF25_pred%_-low and MEF50_pred%_-low groups); and it had the largest decreased fold-difference in MEF50_pred%_-low group (Supplementary Fig. 3). This is in accordance with those studies that reported *P. nanceiensis* as a “beneficial” commensal bacterium in respiratory system. In one of those studies, compared with healthy airways, abundance of *P. nanceiensis* decreased in the airways of patients with chronic obstructive pulmonary disease (COPD), asthma, diabetes, celiac disease, and chronic periodontitis [35, 36]. In another study about children with Henoch-Schönlein Purpura [37], *P. nanceiensis* was observed to be positively correlated with IgA increase. IgA is important at mucosal surfaces for maintaining homeostasis [38, 39], and it complexes activate eosinophils and neutrophils in inflammation. In this situation, *P. nanceiensis* might have participated in the immune responses. This is in accordance with earlier findings that increased *P. nanceiensis* was associated with diminished neutrophilic airway inflammation, suggesting that *P. nanceiensis* is related to Th2-high type asthma [40]. So it is possible that some commensal bacteria of the airways may participate in the regulation of local and distant immune responses [41].

We also observed that some taxa that had negative associations with small airway functions or enriched significantly (*p* < 0.05) in MEF25_pred%_-low or MEF50pred%-low group. Many of these taxa play a role in human lung, oral and cardiovascular diseases [42]. Among these taxa, *C. rectus*, which had the largest negative estimate with all small airway functional indices, was enriched significantly (*p* < 0.05) in MEF25_pred%_-low and MEF50_pred%_-low groups. *C. rectus* was reported to be associated with periodontal disease [43], and was linked to coronary artery disease, lung abscess, empyema, brain abscess, and osteomyelitis [43, 44]. The precise reasons for these associations are unclear. However, evidence showed that *C. rectus* increased production of the proinflammatory cytokines IL-6 and IL-8 in human gingival fibroblasts [45], suggesting it may induce an inflammatory milieu in other tissues.

In this study, *P. nigrescens* was also observed to have a negative estimate with MEF (75/25) and FEV_1_/FVC. More recently, *P. nigrescens* was reported to be associated with signs of carotid atherosclerosis in patients without periodontitis and endodontic infections [46, 47]. The later finding of dental colonization suggests possible distal spread of either the bacteria or inflammatory mediators such as cytokines. Still, patients with asthma show increased risk of bacterial infection. Certain bacterial species may transition from benign to pathogenic activities under some conditions but whether this is true in asthma requires additional research.

*R. dentocariosa* was the only taxa that had negative estimates with all small airway and lung functions observed in this study. *R. dentocariosa* is a normal commensal bacterium of the oral cavity and is associated with dental caries and periodontal disease. The bacterium is also reported to be associated with septic arthritis, pneumonia, arteriovenous infection, and acute bronchitis [48]. Of note, *R. dentocariosa* can upregulation production of TNF-a by T cells [49].

*S. anginosus* was another taxa observed in our study to have a negative relationship with small airway function and it has been reported to be associated with pharyngitis and infections of internal organs and certain body fluids [50].

Functional analysis using PICRUSt showed clear differences between the bacterial predicted metabolic functions in different study group in our work. Pathway analysis of changes in the microbial flora genes indicated that they were related to carbohydrate and amino acid metabolism, cellular processes, and human diseases, and that the changes were distributed in different proportions. These findings are in accordance with other reports and suggest increased metabolic activity of the airway microbiome in asthmatic individuals [51, 52]. However, due to the limitation of PICRUSt, this prediction did not correspond to specific genera. Combining these analytic approaches may yield new insights.

The present study has a number of limitations. First, the cohort sample size is moderate and may not accurately reflect the true population. Second, some important indexes, such as IgA, were not tested for all patients with asthma. Further, the role of seasonal irritants, pollutants and smoke ingestion, such as from tobacco, was not tested in this study.

To sum up, our work gave evidence that small airway function was associated with respiratory tract microbiome, and commensal microorganisms may participate in the regulation of local and distant immune responses. Our findings could provide some information to therapy for patients with “small airway phenotype” asthma.

## Supplementary Information

**Additional file 1: Sup. Table 1.** Experimental materials and reagents. **Sup. Figure 1.** Differences between MEF50 function groups in microbiome composition at species level. **Sup. Figure 2.** Taxonomy tree and LDA scores of the groups. (A) Taxonomy tree and LDA scores between MEF50predicted%-low and MEF50predicted%-high groups. (B) Taxonomy tree and LDA scores between the MEF50predicted%-low group and the healthy control group. Circles from within to outward indicate the classification from the phylum to the genus, respectively. Each small circle represents a taxon with its diameter proportional to the relative abundance. Dots with different colors denote the core species of each group. Histogram showing the LDA scores of the biomarkers with statistical differences. **Sup. Figure 3.** Volcano plot of fold change (≥ 1.2-fold, adjusted *p* < 0.05) between the MEF_50predicted%_-low and MEF_50predicted%_-high groups in subjects with asthma.

## Data Availability

The 16S RNA datasets generated and analyzed during the current study are now accessible in the NCBI repository: https://www.ncbi.nlm.nih.gov/bioproject/PRJNA879958.

## Abbreviations

ACQ6: Asthma Control Questionnaire 6
BMI: Body Mass Index
GINA: Global Initiative for Asthma
FeNO: Fractional Exhaled Nitric Oxide
FEV1: Forced Expiratory Volume in 1 s
FVC: Forced Vital Capacity
MEF25: Maximal Expiratory Flow in 25% Vital Capacity
MEF50: Maximal Expiratory Flow in 50% Vital Capacity
MEF75: Maximal Expiratory Flow in 75% Vital Capacity
MEF_25pred%_: Percentage of Tested MEF25 of Predicted MEF25
MEF50_pred%_: Percentage of Tested MEF50 of Predicted MEF50.
PCA: Principal Component Analysis
PEF: Peak Expiratory Flow Rate
KEGG: Kyoto Encyclopedia of Genes and Genomes
LEfSe: Linear Discriminant Analysis Effect Size method
PLS-DA: Partial Least Squares Discriminant Analysis
PICRUSt: Phylogenetic Investigation of Communities by Reconstruction of Unobserved States
VC: Vital capacity
