# Supplementary material for: Upper respiratory tract microbiota is associated with small airway function and asthma severity
Source: BMC Microbiol. 2023 Jan 13;23:13. doi: 10.1186/s12866-023-02757-5 (PMC9837891; doi:10.1186/s12866-023-02757-5)
Supplement: Supplementary file 1 — Additional file 1: Sup. Table 1. Experimental materials and reagents. Sup. Figure 1. Differences between MEF50 function groups in microbiome composition at species level. Sup. Figure 2. Taxonomy tree and LDA scores of the groups. (A) Taxonomy tree and LDA scores between MEF50predicted%-low and MEF50predicted%-high groups. (B) Taxonomy tree and LDA scores between the MEF50predicted%-low group and the healthy control group. Circles from within to outward indicate the classification from the phylum to the genus, respectively. Each small circle represents a taxon with its diameter proportional to the relative abundance. Dots with different colors denote the core species of each group. Histogram showing the LDA scores of the biomarkers with statistical differences. Sup. Figure 3. Volcano plot of fold change (≥ 1.2-fold, adjusted p < 0.05) between the MEF50predicted%-low and MEF50predicted%-high groups in subjects with asthma. [file 12866_2023_2757_MOESM1_ESM.docx]

Table 1. Experimental materials and reagents

| **Reagemt** | **Type** | **Producer** | **country** |
| --- | --- | --- | --- |
| DNA Extraction Kit | FastDNA Spin Kit for Soil | MP Biomedicals | U.S.A. |
| agarose |  | Biowest | Spain |
| FastPfu Polymerase | FastPfu Polymerase | TransGen | China |
| AxyPrep DNA Gel Extraction Kit | Axygen Biosciences | Axygen | U.S.A. |
| DNA-library Kit | NEXTFLEX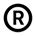 Rapid DNA-Seq Kit | [Bioo Scientific](http://www.baidu.com/link?url=BRWnmmM9J_KdHms5U2w7PjsETSlWBUzBlZ_uTrItjmXv_FeJdly65jT2x5rCN5rRFvrgrGluiOIym-OxWFSfb6Tr5V4UcrS3rGmZHNwMD6q) | U.S.A. |
| DNA-Sequencing Kit | MiSeq Reagent Kit v3 | Illumina | U.S.A. |


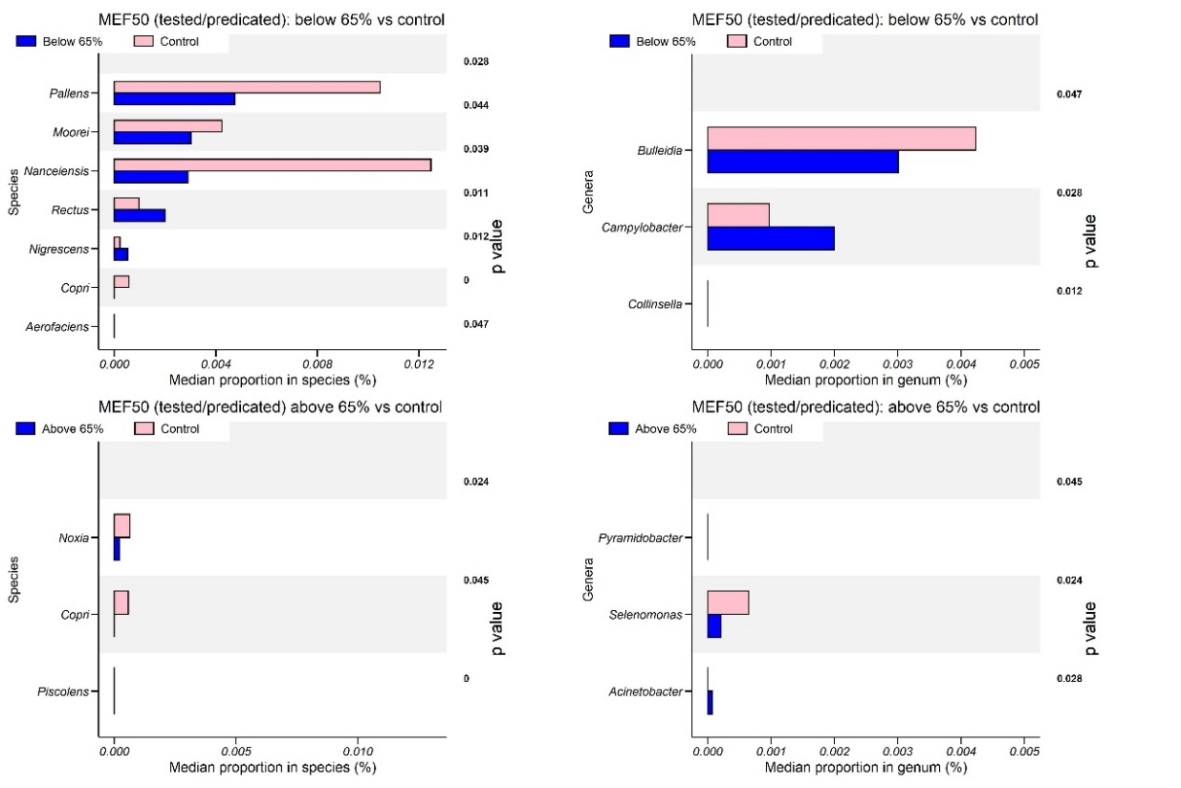


Sup. Fig 1. Differences between MEF50 function groups in microbiome composition at species level


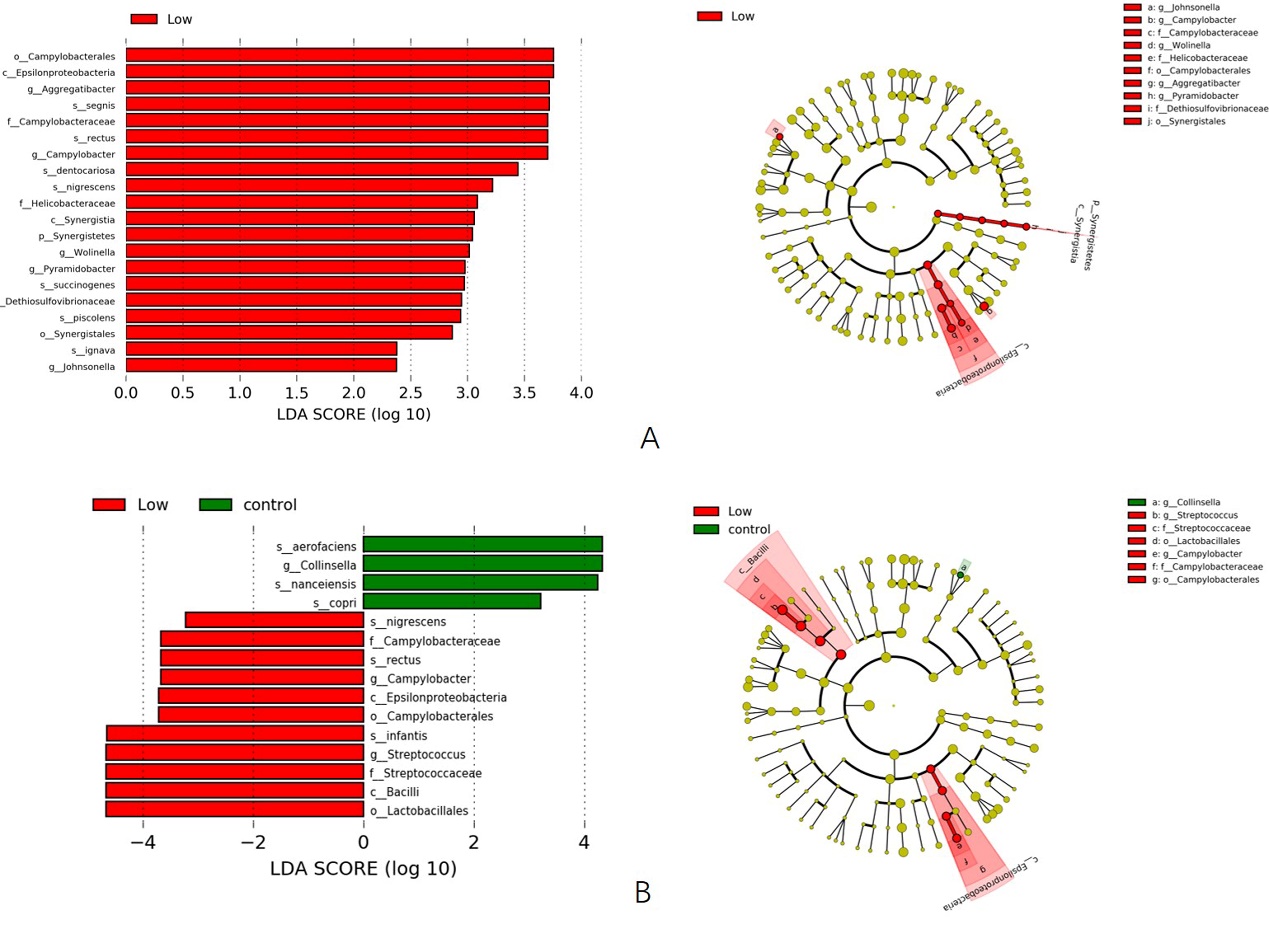


Sup. Fig 2. Taxonomy tree and LDA scores of the groups. (A) Taxonomy tree and LDA scores between MEF50predicted%-low and MEF50predicted%-high groups. (B) Taxonomy tree and LDA scores between the MEF50predicted%-low group and the healthy control group. Circles from within to outward indicate the classification from the phylum to the genus, respectively. Each small circle represents a taxon with its diameter proportional to the relative abundance. Dots with different colors denote the core species of each group. Histogram showing the LDA scores of the biomarkers with statistical differences.


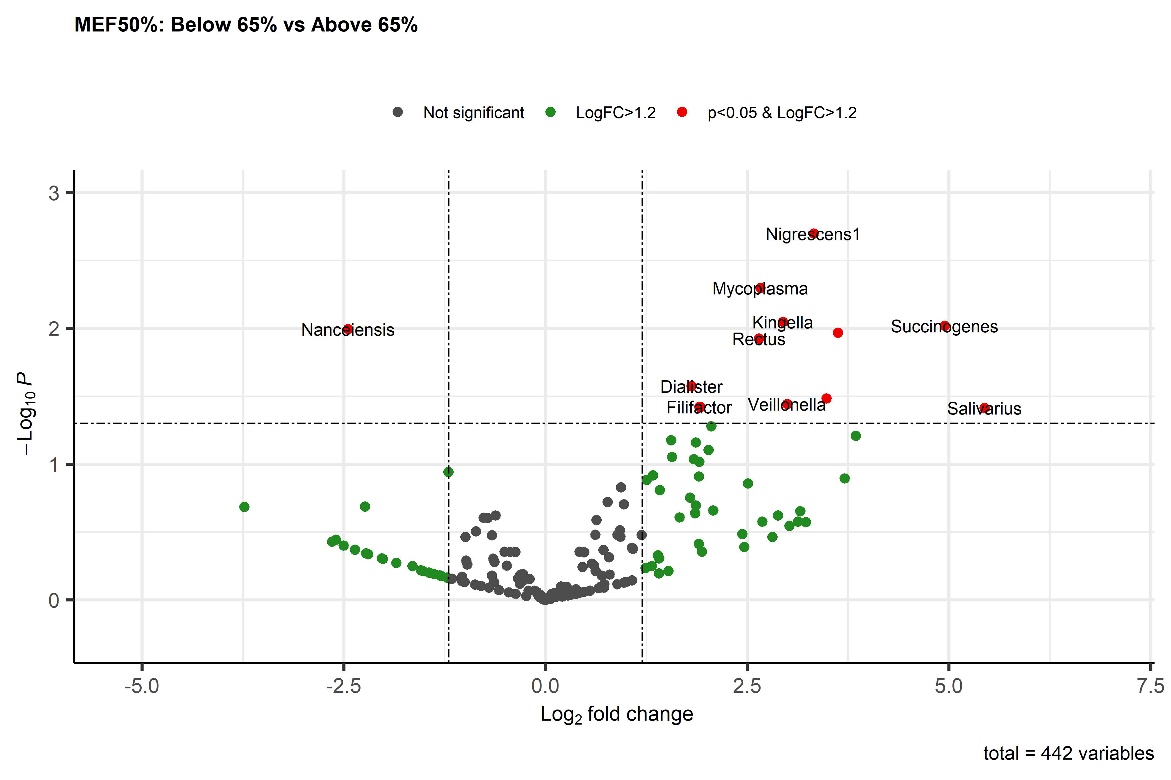


Sup. Fig 3. Volcano plot of fold change (≥ 1.2-fold, adjusted p < 0.05) between the MEF_50predicted%_-low and MEF_50predicted%_-high groups in subjects with asthma. Dashed lines indicate 1.2-fold difference and an FDR significance level of 0.05%.
